# Airports for the genetic rescue of a former agricultural pest

**DOI:** 10.1038/s41598-024-68679-y

**Published:** 2024-07-30

**Authors:** Matúš Búci, Diana Krajmerová, Branislav Tám, Peter Kaňuch, Peter Klinga

**Affiliations:** 1 Institute of Forest Ecology, Slovak Academy of Sciences, Zvolen, Slovakia; 2 Faculty of Ecology and Environmental Sciences, Technical University in Zvolen, Zvolen, Slovakia; 3 Faculty of Forestry, Technical University in Zvolen, Zvolen, Slovakia; 4 National Zoo Bojnice, Bojnice, Slovakia; 5 Faculty of Forestry and Wood Sciences, Czech University of Life Sciences Prague, Prague, Czech Republic

**Keywords:** Ecological genetics, Conservation biology

## Abstract

The intensification of agricultural practices and urbanisation are widespread causes of biodiversity loss. However, the role of artificial habitats in genetic rescue is an aspect that is not well understood. Implementing genetic rescue measures to improve gene flow and maintain a viable population of keystone species is a crucial prerequisite for promoting diverse and resilient ecosystems. Landscape fragmentation and modern agricultural methods have caused the decline and the isolation of the remnant colonies of the endangered European ground squirrel (*Spermophilus citellus*) throughout its range. However, the artificial habitat, such as airport fields with regular grass mowing, provides suitable conditions for this grassland specialist. We measured home range size and genetic variation of seven souslik colonies in western Slovakia. Based on the 6904 ddRAD SNPs, we found significantly higher individual heterozygosity in colonies on airports compared to colonies on pastures. This indicates a potential for higher fitness of individuals from airport colonies, which can serve as a source for evidence-based translocations. Such an intervention can preserve the genetic diversity of small and isolated populations in the region. We emphasize that conservation management strategies would be strengthened including a specific focus on human-made grassland habitats.

## Introduction

Habitat loss and fragmentation are the greatest threats to biodiversity. The exponential growth of human activities with the intensification of agricultural practises and urbanisation leads to the loss of natural and semi-natural areas and has consequences for the distribution and survival of species^1^. Animal populations are affected either directly (e.g., through reduced food availability, restricted movement opportunities) or indirectly through limited gene flow and thus evolutionary processes that affect genetic and phenotypic traits, animal behaviour or ability to adapt to a changing environment^2^. Genetic erosion refers to the loss of genetic diversity in a population over time and can reduce fitness and ultimately contribute to population extinction^3,4^. Furthermore, genetic drift and inbreeding influence genetic patterns in small fragmented populations^5^. All this leads to a decrease in genetic diversity and an increase in genetic differentiation between isolated populations^6,7^. In the short term, inbreeding can increase the expression of deleterious recessive alleles, resulting for example, in increased mortality of juveniles^8^. In the long term, genetic erosion can reduce the resilience of species and communities to environmental stress^9^.

Genetic diversity is fundamental to the process of natural selection. Understanding how genetic diversity correlates with individual fitness is essential for predicting evolutionary processes^10^. The heterozygosity-fitness correlation (HFC) correlates the level of individual genetic diversity with fitness-related traits^11,12^. The advantage of individual heterozygosity lies in the ability to suppress deleterious alleles (dominance) instead of creating overdominance^13^. Parental genetic diversity can, for example, influence the fitness of their offspring through different fertilisation success^14^, immunity^15^ or through the different quality of parental care^16^. Therefore, an estimate of individual heterozygosity can be used to solve genetic-based conservation problems. Individual heterozygosity is a strong predictor of survival potential and is unrelated to read depth, sample age, the influence of outlier loci and library complexity^17^.

In an effort to prevent the extinction of declining fragmented populations, reintroductions can serve as an effective conservation tool^18^. The main purpose is to translocate a group of individuals from a stable population (wild or captive) to historical areas of the species distribution range in order to establish or augment an existing population^19,20^. Conservation translocations can integrate new alleles into genetically eroded populations, resulting in changes in allele frequencies and an enhancement in genetic diversity and population growth^21^. The optimal strategy for increasing fitness and evolutionary potential in rescued populations is to ensure that the source populations have the broadest possible range of evolutionary diversity. This maximises genetic variation within the target population, which promotes resilience and adaptability in the face of environmental challenges^22,23^. Thus, gene flow from genetically diverse populations is better able to reverse genetic erosion than that gene flow from small populations^23^. However, selecting partially-inbred genetic sources for genetic rescue, e.g., from captivity, can be detrimental^20^. Similarly, if the local adaptations are present in both the source and recipient populations, translocations can be counterproductive, as outbreeding depression can reduce population viability^24,25^.

In a rapidly changing global environment, genetic rescue of populations holds relevance for potentially any species, even those previously categorised as common, abundant or even pests. For example, there are 14 species of Old World ground squirrels (genus *Spermophillus*), including several subspecies^26^, distributed mainly in China, Mongolia, Russia, Central Asia and Asia Minor^27^. During the period of agricultural intensification up to the end of the twentieth century, these rodents were considered important pests and causative agents of zoonoses, e.g., *Yersina pestis*, *Salmonella enteritidis*, *Francisella tularensis*^28–30^ in some regions. The use of pesticides to control plague outbreaks has eradicated some populations or reduced their natural range^31^. However, populations of ground squirrels in Europe have been decimated in recent decades, mainly through habitat loss and modern agricultural practises^32^. For this reason, the European ground squirrel or souslik (*Spermophilus citellus*) is now a key endangered species of grassland-steppe ecosystems in Central and South-eastern Europe^33^. In the absence of suitable habitats such as grazed grassland or pastures, the island-like distribution of souslik colonies is currently concentrated on the airport environment surrounded by arable land^34^.

Although airports attract attention mainly because of the risk of collisions between wildlife and aircraft^35,36^, this artificial habitat can also be important for the conservation of biodiversity. There is a paucity of evidence to suggest that airports play a positive role in mammal conservation, conversely there is a potentially large but equally under-researched capacity for sousliks in Europe. Airports provided an alternative refuge for disappearing souslik colonies, which are threatened by the conversion of pasture habitats into arable land. In spite of several translocation attempts to establish new populations carried out since 2000, only the translocation from Bratislava International Airport to the natural landscape in western Slovakia (Kuchyňa), can be considered a success, as it persisted more than 15 years since the release of the last individual^37^. The lack of genetic evidence about the founders and the genetic composition of the recipient colonies, combined with insufficient habitat management, are thought to be the reason for the low success rate. Thus poor information about genetic variation in the species of conservation concern can undermine the effectiveness of conservation actions^2^. In this study, we therefore aimed to explore the genetic diversity of isolated souslik colonies in Western Slovakia, which is at the northern edge of the species’ European range. The objectives were: (1) to measure the home range of colonies and to assess the potential for range expansion in relation to the availability of grassland habitats in the surrounding area; and (2) to compare estimates of individual heterozygosity in individuals from different habitats to determine whether populations inhabiting an airport fields might be a potentially suitable source of genetic rescue for souslik colonies in a fragmented landscape.

## Results

**Figure 1:**
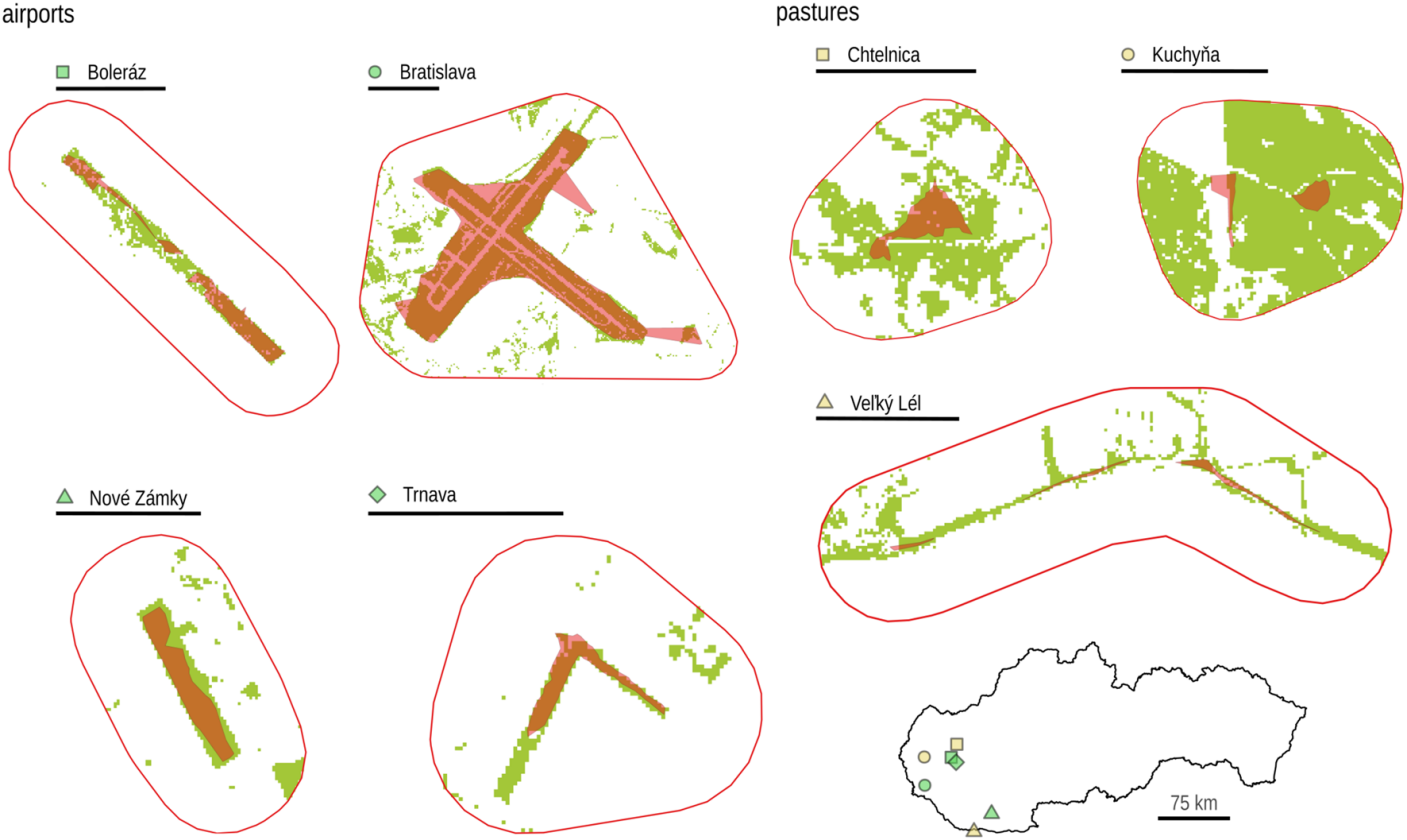
Reference source not found. Home ranges (red polygon) of *Spermophilus citellus* colonies and suitable grassland habitats (green) within the buffer zone of their potential expansion at different sites in western Slovakia. The scale bars at each site correspond to 1 km.

The mean size of the colonies’ home range was 0.66 km^2^ (range 0.04–3.93 km^2^). Based on the higher proportion of available grassland within the buffer zone for colonies on pastures (81–97%) compared to airports (29–60%), we suggested that the expansion potential of colonies on pastures was relatively greater than on airports (Table 1). Thus, airport fields were mostly surrounded by arable land which was unsuitable for souslik (Fig. 1).

**Table 1:** Colonies of *Spermophilus citellus* in western Slovakia at airports and pastures.

Site	WGS84 coordinates	CS	*n*	Home range (km^2^)	Buffer (km^2^)	G_T_ (%)	G_A_ (%)
Airports							
Boleráz	48.453 N, 17.523 E	600	5	0.23	4.1	10	56
Bratislava	48.170 N, 17.212 E	7000	10	3.93	16.4	22	29
Nové Zámky	47.962 N, 18.184 E	600	8	0.16	2.2	15	53
Trnava	48.401 N, 17.608 E	100	2	0.07	2.0	7	60
Pastures							
Chtelnica	48.572 N, 17.602 E	100	3	0.10	1.8	37	81
Kuchyňa	48.423 N, 17.163 E	200	8	0.06	2.1	73	97
Veľký Lél	47.760 N, 17.975 E	50	10	0.04	4.2	11	93

*CS* estimated colony census size (number of active burrows), *n* sample size; the home range of the colony and the buffer zone, *G*_*T*_ total proportion of grassland within the buffer zone, *G*_*A*_ proportion of available grassland area outside the colony’s home range within the buffer zone.

**Figure 2:**
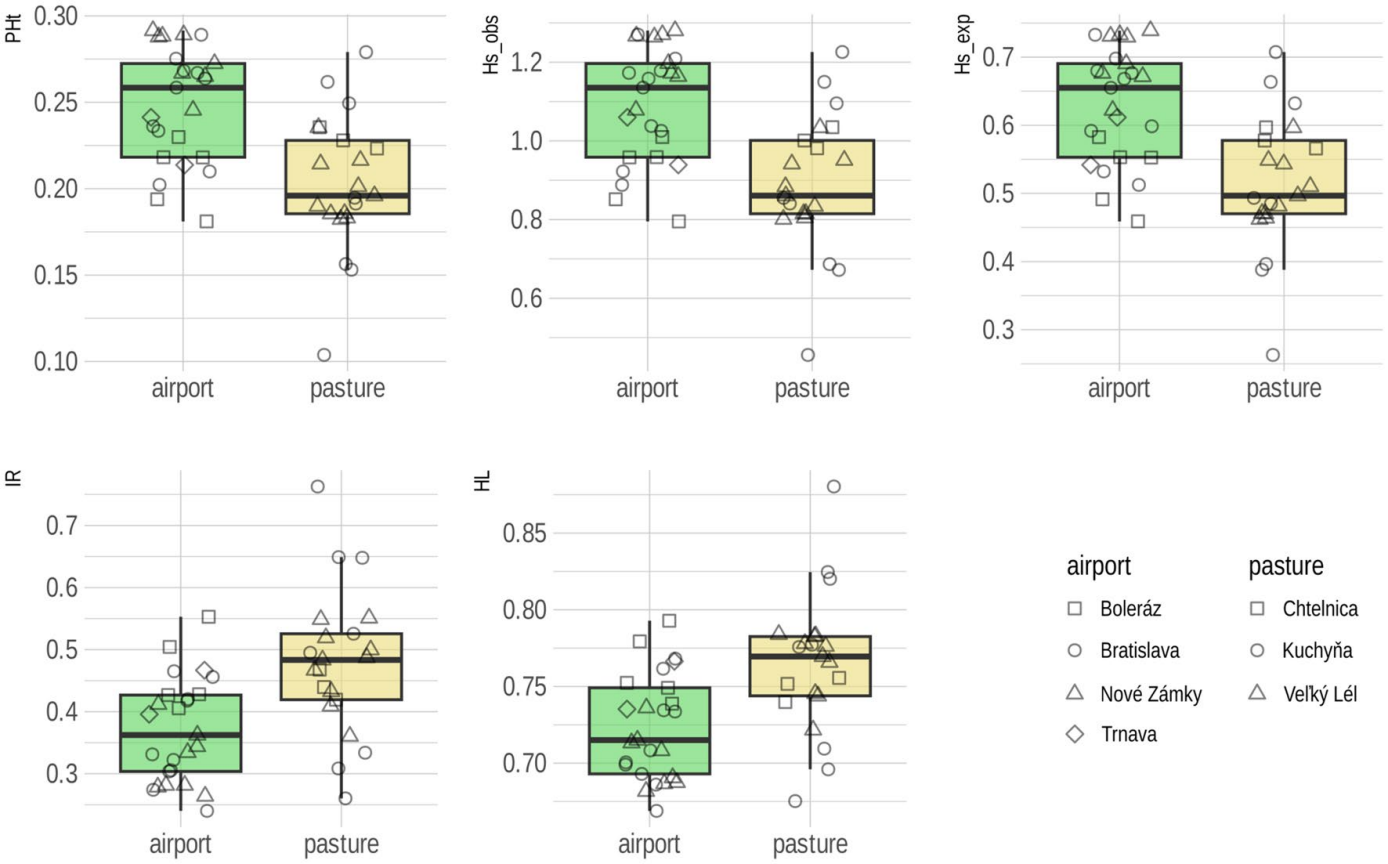
Reference source not found. Indices of individual heterozygosity of *Spermophilus citellus* (*PHt* proportion of heterozygous loci, *Hs_exp* standardised heterozygosity based on the mean expected heterozygosity, *Hs_obs* standardised heterozygosity based on the mean observed heterozygosity, *IR* internal relatedness, *HL* homozygosity by locus) in different habitats. Symbols indicate the site of origin of the individuals.

The analysis of 46 individuals at 6,904 SNPs revealed an average proportion of heterozygous loci (*PHt*) of 0.228 (range 0.199–0.276), standardised heterozygosity based on the mean observed heterozygosity (*Hs_obs*) of 1.000 (0.873–1.212), standardised heterozygosity based on the mean expected heterozygosity (*Hs_exp*) of 0.577 (0.504–0.699), internal relatedness (*IL*) of 0.421 (range 0.320–0.498) and homozygosity by locus (*HL*) of 0.742 (0.702–0.770). Individuals living in airports had significantly (p < 0.001) higher values of *PHt*, *Hs_obs* and *Hs_exp* and significantly (p < 0.001) lower values of *IR* and *HL* than individuals originating from colonies on pastures (Fig. 2).

## Discussion

Artificial habitat structures are purpose-built, man-made substitutes for natural habitats designed to support the conservation of animals by improving survival, growth, reproduction and abundance. Building on this definition, we show that artificial habitat structures designed and managed for transport infrastructure purposes can also serve as substitutes for natural habitat structures in degraded, disturbed or altered environments.

In the present study, we found that sousliks living in airports exhibit higher heterozygosity than individuals living in pastures, which unlike the former, have a higher potential for spatial dispersal due to suitable grassland habitats in their environment. The higher individual heterozygosity could be explained by local demography as the airport colonies generally have larger numbers of individuals (Table 1), suggesting a higher recombination rate that increases variability within the population. The central range colonies in Romania, Hungaria and Austria have been recognised as genetically more diverse than colonies living at the edge of the species distribution in Czechia^38^. In our study area, human intervention to souslik habitats and populations through past translocations from core to peripheral populations might outperform possible edge effect of fragmented populations. Individual heterozygosity is a crucial and easily quantifiable indicator for predicting the survival of populations threatened with extinction and/or undergoing a translocation programme, eclipsing the importance of population size and overall diversity^17^. Many studies on heterozygosity-fitness correlations, including rodent species (e.g.^39,40^), have shown higher reproductive success^41^, higher disease resistance^42^ and higher survival probability of individuals with higher heterozygosity^17^. Genetically diverse populations have greater evolutionary capacity to respond to ecological stressors than small, inbred populations with a higher frequency of slightly deleterious mutations that manifest in homozygotes^43^. Considering these facts, we could assume that individuals from the airport colonies in western Slovakia can also have a higher fitness compared to populations surviving in other isolated semi-natural habitats. Endangered mammal species are usually associated with anthropogenic landscape fragmentation^44^. Such species are often poorly adapted to the human-altered environment^45^. However, as grassland specialists, sousliks find ideal habitat conditions at airports^34^. At the airports in our study, sousliks live in regularly mown grasslands, which could be linked to the availability of food resources, the minimal chemical treatment of the soil and the lower risk of predation. To date, very few studies have been conducted on species conservation at airports (e.g.^45^). The role of airports in biodiversity conservation has been little analysed in the literature and has been evaluated more negatively than positively^46^. In contrast, our results emphasise the importance of artificial landscapes such as airports for the conservation of biodiversity on intensively farmed land. However, the persistence of souslik colonies at airports attracts birds of prey thereby increasing the risk of aircraft collisions. This poses a significant threat to aviation safety, rendering the presence of souslik at airports undesirable. Furthermore, while the costly management of these areas combined with the high risk of aircraft collisions would probably outweigh conservation interests.

In a human-altered environment where natural gene-flow is unlikely, as in the case of souslik colonies in grazed habitats in western Slovakia, translocations can lead to the overall maintenance of their genetic diversity^47^. The processes of eliminating the causes of population decline and ensuring the suitability of the habitat should precede any translocation attempts^48^. To maximise the success of translocations, it is recommended to transfer individuals multiple times (e.g., annually) with a number of founders that covers ≥ 95% of the genetic diversity of the source population^49^. However, environmental variables such as food availability or soil temperature can have a significant impact on local adaptations^50^. For example, in the Columbian ground squirrel (*Urocitellus columbianus*), differences in the timing of torpor emergence were found after translocating individuals to populations with different phenology^51^. To increase the genetic variability of colonies living in pastures, we therefore suggest translocating individuals from airports within the same region, taking into account their local adaptations. In recent decades, hasty pest control decisions driven by economic interests have often led to poor management of target species and/or threats to biodiversity^52^. The recovery of a former agricultural pest can potentially develop into a conflict between agriculture and wildlife^53^. However, ecologically-based rodent management could predict species movement and densities, allowing earlier and more effective interventions that minimise uncontrolled biodiversity damage and crop losses^54^. In addition to saving genetically eroded colonies, such measures could also be beneficial for airport management to control rodent density there. The findings suggest that understanding the positive and negative effects of anthropogenic landscapes on biota can play an important role in the conservation and management of species living in heavily modified environments.

In conclusion, high-resolution genomic datasets have revealed the important role of airport ecosystems in conserving and maximising the genetic diversity of an endangered species. The results of our study highlight the potential of airports as valuable sources for genetic rescue programmes targeting the European ground squirrel in the region. We strongly advocate urgent genetic rescue initiatives for colonies persisting on isolated pastures and emphasise the clear benefit of including individuals from airport ecosystems in reintroduction programmes. This admixture strategy will likely optimise genetic diversity and can enhance the adaptive potential within genetically eroded colonies in natural habitats.

## Materials and methods

### Study area and souslik colonies

The study area in western Slovakia belongs to the Pannonian bio-geographical region (Podunajská nížina and Záhorská nížina lowlands) with moderate warm and warm climate (the annual mean temperature is − 2 °C in winter and 25 °C in summer, annual precipitation below 500 m). The original grassland and steppe habitats were largely transformed into agricultural land at the end of the twentieth century. In this area, we surveyed grassland habitats of all known colonies whose occurrences were recorded as part of the species’ monitoring programme in 2018–2019. Thus, we sampled seven souslik colonies inhabiting two different habitats. Four colonies inhabited airport fields and three colonies occurred on pastures (Table 1). The altitude of the colonies was between 112 and 300 m.a.s.l. Paired geographical distances between colonies ranged from 7 to 72 km, which exceeds the species’ maximum dispersal distance of up to one kilometre^55^, and we are confident that there is no natural gene flow between these colonies (own unpublished results of a Structure analysis).

### Data collection and home range estimation

In each colony, we recorded the coordinates of all burrows using transect method frequently used for abundance and population density assessment of the *Spermophilus* species^56–58^. The colony size was estimated based on the number of active burrows (recognisable by the excavated fresh soil in front of the burrow). To determine the home range size for each colony, we calculated a local convex hull nonparametric kernel area (LoCoH) with a parameter range of 600–3500 according to the recommendations of Ref.^59^ using the ‘AdehabitatHR’ package^60^ of the R 4.1.2 software^61^. The minimum convex polygon (MCP) method was used to measure the buffer zone, which was set at 500 m from the home range, which is beyond the average dispersal ability of the species (350 m)^62^. The proportions of home ranges and grassland habitats based on the High Resolution Layer Grassland 2015–2018 (https://land.copernicus.eu/en/products/high-resolution-layer-grassland) within the buffer zone were visualised and measured using the QGIS 3.34 programme (https://qgis.org).

For genetic samples, individuals were caught in box traps during their active season (from April to September) in 2019 and 2020^37^. In total, we collected 46 tissue samples (25 ind. from four airports, 21 ind. from three pastures), with the size of the samples reflecting the local population census size and/or the range of the colony (Table 1). The captured adults were assigned with the geographical coordinates of their capture and sampled by clipping a 0.5 cm long tail segment. We sampled individuals in the same sex ratio to avoid possible bias due to the sex-specific dispersal behaviour of males and females in the wild. Box traps were systematically distributed around the colony at the most distant locations to minimise the capture of closely related individuals. Tissue samples were stored in 96% ethanol and cooled in a freezer at − 20 °C until DNA extraction. Each captured individual was released back into the wild.

### DNA extractions, genotyping and statistics

For DNA extraction, we used the commercial GeneJET FFPE DNA Purification Kit (ThermoFisher Scientific) according the protocol optimised for DNA extraction from animal tissue samples. The genomic library was assembled using the ddRAD sequencing protocol^63^. Suitable combination of restriction endonucleases (Sphl, EcoRI) was selected based on the results of ‘in silico’ analysis of related species.

The ddRAD sequencing was performed by the IGA Technology Services (Italy) in pair-end mode using a Novaseq6000 sequencer (Illumina, San Diego, CA). The DNA sequences were aligned with the reference genome of the Daurian ground squirrel (*Spermophilus dauricus*) using the BWA (Burrows–Wheeler Alignment Tool) add-on of Stacks 2.0 software^64^. The GSTACKS add-on software of Stacks was used to detect all genomic DNA fragments (all aligned loci). In the next step, the fragments or loci were filtered using the POPULATION programme implemented in Stacks. Single nucleotide polymorphisms (SNPs) were filtered based on the following criteria: quality value Q > 13 (in our case Q = 26 was the lowest true value, thus, no SNPs were removed), missing data in more than 20% of the samples (12%; 4231 markers were removed; 31,128 out of 35,359 markers were retained), less than 5% minimum allele frequency (39.9; 12,406 markers were removed; 18,722 of 31,128 markers were retained) and a read depth of ± 2 standard deviations (4.8%; 906 markers were removed; 17,816 of 18,722 markers were retained). The last step was to remove SNPs that are potentially in linkage disequilibrium, so that finally 6904 SNPs were used to analyse genetic variation.

The potential bias resulting from the small sample size per site is minimised by using an individual-based approach that includes individuals from all known colonies in both natural and airport habitats within this part of the species’ range. Five estimates were used to calculate individual heterozygosity: proportion of heterozygous loci (*PHt*), standardised heterozygosity based on the mean observed heterozygosity (*Hs_obs*), standardised heterozygosity based on the mean expected heterozygosity (*Hs_exp*), internal relatedness (*IR*) and homozygosity by locus (*HL*) using the R function GENHET^65^. The difference between the habitat types (airport vs. pasture) was determined for each estimate using the non-parametric Wilcoxon rank-sum test implemented in the base ‘stats’ package of R 4.1.2 software^61^.

### Ethics approval

This study adhered to the Guidelines for the Treatment of Animals in Behavioral Research and Teaching^66^ and the legal requirements of the country (Slovakia) in which the work was performed. The protocol for this research was approved by the Ministry of the Environment of the Slovak Republic (5107/2019-6.3) according to the decision of its own ethics review board. ARRIVE guidelines for reporting animal research have been followed as much as possible (https://arriveguidelines.org/).

## Data Availability

Sequences analysed during the current study are available in SRA database of the National Center for Biotechnology Information (NCBI), BioProject Accession Number PRJNA1131305 https://www.ncbi.nlm.nih.gov/sra/PRJNA1131305.
